# Quick question or intensive inquiry: The role of message elaboration in the effectiveness of self-persuasive anti-alcohol posters

**DOI:** 10.1371/journal.pone.0211030

**Published:** 2019-01-24

**Authors:** Jeroen G. B. Loman, Sarah A. de Vries, Niels Kukken, Rick B. van Baaren, Moniek Buijzen, Barbara C. N. Müller

**Affiliations:** 1 Behavioural Science Institute, Radboud University, Nijmegen, Gelderland, The Netherlands; 2 Institute of Cognitive and Evolutionary Anthropology, University of Oxford, Oxford, United Kingdom; 3 Social Cognition and Decision Sciences, Eberhard Karls Universität Tübingen, Tübingen, Baden-Württemberg, Germany; Univerity of Salzburg, AUSTRIA

## Abstract

Self-persuasion (i.e., generating your own arguments) is often more persuasive than direct persuasion (i.e., being provided with arguments), even when the technique is applied in media messages by framing the message as a question. It is unclear, however, if these messages are more persuasive when viewed for a long period to allow more elaboration about the message, or for a short period to reduce elaboration. In the current experiment, this is addressed by examining whether anti-alcohol posters framed as a statement (direct persuasion) or an open-ended question (self-persuasion) are more effective to reduce alcohol consumption under conditions of short- or long message exposure, compared to a control condition (no poster). Additionally, the potentially moderating roles of self-perceived alcohol identity and self-esteem on both types of persuasion are examined. Participants (*N* = 149) were exposed to a self-persuasion or direct persuasion anti-alcohol poster, either briefly before or continuously during a bogus beer taste task. The amount of alcohol consumed was the covert dependent variable. Contrary to expectations, both posters failed to affect alcohol consumption, regardless of exposure length. No moderation effects for self-perceived alcohol identity and self-esteem of the participants were found. Possible explanations are discussed.

## Introduction

Alcohol consumption is one of the major avoidable risk factors contributing to global disease and death [1–3]. Despite numerous media interventions aiming to reduce consumption, in the majority of countries drinking levels remain stable or continue to rise [4,5]. Recent literature investigating the effectiveness of anti-alcohol media messages to change antecedents of drinking or actual alcohol consumption behavior yielded mixed results. Some research found that anti-alcohol media messages were effective in reducing the urge to drink [6] or intentions to binge drink [7]. Other studies found no differences in intentions to drink after viewing anti-alcohol advertising versus alcohol advertising [8], and no differences in actual consumption after viewing anti-alcohol posters compared to no persuasion controls [9]. Again other studies have found that anti-alcohol media messages resulted in less negative attitudes towards alcohol [10], or even led to greater alcohol consumption [11].

Overall, there is little scientific research available regarding the effectiveness of counter-advertising and media advocacy, and that which is available also shows inconclusive results ([12–15]; see also [16]). Part of the problem is that media campaigns that promote responsible drinking are ineffective compared to the much larger number of high-quality pro-drinking advertisements in mass media. In light of these findings, and from the perspective of persuasive communication, serious efforts should be made to test alternative and innovative persuasion methods to increase the effectiveness of anti-alcohol media interventions.

Research suggested that part of the reasons why media interventions have been largely unsuccessful is because anti-alcohol media messages typically consist of direct forms of persuasion (i.e., *providing* arguments or statements), which are rather ineffective [17]. Instead, it has been suggested that self-persuasion techniques (i.e., using open-ended questions to have individuals *generate* arguments themselves) could have some (albeit small) persuasive benefit over direct methods ([18–20]; also see [17]). Though these findings are interesting, it should be noted that most of these studies (i.e., [18–20]) have low power and should therefore not be interpreted as conclusive evidence. Because of this, further testing seems appropriate.

When considering the application of self-persuasive media messages in real-life, it is important to know if persuasion occurs after long exposure, allowing message receivers to elaborate about the message, or after short exposure to reduce elaboration. This information could aid interventions to find suitable outlets for self-persuasive media messages, such as posters in bars (long exposure) versus commercials on television (short exposure). The current experiment answers this question by examining the role of message elaboration in relation to direct- and self-persuasive anti-alcohol posters that are designed to reduce alcohol consumption.

### Self-persuasion

Self-persuasion techniques (see [21]) rely on individuals to think of arguments to do (or not do) something, in order to persuade themselves. In other words, the targets of persuasion create the means of influence themselves [22,23]. This type of persuasion is highly effective and has been studied extensively. Notable examples include opinion change resulting from presenting a talk in favor of some topic [24], having people write essays [25], or arguments [26] to (not) do something (for a more detailed overview, see [17]). More recent studies show that self-persuasion can even be applied successfully in persuasive media messages by framing the message as a question, which is assumed to triggers argument generation in the message receivers [17,20].

From a persuasive media standpoint, self-persuasion has three distinct advantages over more commonly used direct forms of persuasion in which information is provided. First, people mentally detect and correct information that is generated internally less than information that is provided externally [27,28]. Second, psychological reactance is not activated when people generate their own arguments, because their freedom to choose is not restricted. Third, people tend to come up with reasons that they find the most compelling when they generate arguments, which effectively results in self-tailoring the most persuasive message possible for themselves [22,29,30].

Overall, the application of self-persuasion techniques in persuasive media messages seems promising. Not only do self-persuasion methods seem more effective to change attitudes and behavior than direct forms of persuasion, they are also easily applicable in mass media messages, which have the potential to reach very large audiences. Although self-persuasion is researched in different kinds of settings (e.g., giving speeches [31]; listing arguments [32]; or in conversations [33]), its application in media messages is still relatively new, which means important questions about the conditions in which self-persuasive media messages are most effective are yet to be addressed. To date, the most notable examples of self-persuasion in media messages have focused on whether or not the messages are effective to change attitudes, cognitions and behaviors [17–20]. In these experiments however, exposure to the messages was longer and more explicit than might be the case in real life where media messages are often seen briefly. As a result, participants in these experiments were likely to have thought more and longer about the messages than they would have in a real-life situation, which could have affected self-persuasion effects. To better understand whether the application of self-persuasion in media interventions is viable in real life, a logical next step is to examine the effects of message elaboration on self-persuasion [17].

### Message elaboration

Message elaboration is the extent to which people think consciously about a message [34]. For self-persuasive media messages this is important, because in order to persuade, some elaboration is required to generate arguments. However, it is unknown if persuasion increases when elaboration increases. On the one hand, high message elaboration will result in more generated thoughts [35] and therefore more self-generated arguments. Given that more arguments increase persuasion ([36–38]; also see [22]), one would expect self-persuasive media messages to be most effective when elaboration is high. There are two reasons to expect, however, that the messages might be more persuasive when message elaboration is low.

First, self-persuasion research has shown that generating a small number of arguments can be more persuasive than generating a large number of arguments [39]. The explanation is that generating a small number of arguments is easier than generating many. In turn, this feeling of ease is more important than the number of produced arguments, because it feels more ‘fluent’ [40], which renders the message more persuasive.

Second, when individuals see a self-persuasive message briefly, they are likely to automatically generate arguments in response to the question in the message [17]. As message elaboration increases, however, it becomes more likely that message receivers will start to generate counter-arguments for the target behavior [35,41,42], which could render the messages less persuasive or even ineffective. Therefore, conditions of low message elaboration should increase the persuasiveness of self-persuasive media messages. For direct persuasion, similar effects are expected.

### The moderating role of message receiver characteristics

Message receiver characteristics could moderate the effectiveness of self- and direct persuasion. Two characteristics pertaining to the strength and relevance of anti-alcohol appeals are considered here. The first is self-esteem [43]. Because self-persuasion relies on message receivers to generate arguments to convince themselves, higher levels of self-esteem might result in increased confidence in the validity of the arguments, which in turn increases self-persuasion [44]. Conversely, for direct persuasion higher levels of self-esteem are likely to decrease external persuasion [45] and increase reactance responses [46]. The second receiver characteristic is alcohol identity (i.e., the extent to which someone considers alcohol as important for their identity; [47]). The more important alcohol is for an individual, the more relevant anti-alcohol messages are, which increases message relevant thought and decreases the likelihood the messages will be ignored [48,49], possibly increasing self-persuasion due to argument generation and decreasing direct persuasion due to defensive responses.

### The current experiment

The current experiment aims to test the effectiveness of anti-alcohol posters framed as open-ended questions (self-persuasion) or statements (direct persuasion) to reduce alcohol consumption in a beer taste test, under conditions of short- (to manipulate low message elaboration) and long- (to allow high message elaboration) message exposure, compared to a control condition without a poster. Additionally, the moderating roles of self-perceived alcohol identity and self-esteem of the participants on both types of persuasion will be examined.

It is hypothesized that self-persuasion will be more effective to reduce alcohol consumption compared to direct persuasion and no persuasion. Additionally, both self- and direct persuasion are expected to decrease alcohol consumption more under conditions of low message elaboration compared to high message elaboration. Differential effects for both persuasion techniques are expected based on the message receiver characteristics self-esteem and self-perceived alcohol identity. Specifically, self-persuasion is hypothesized to be more effective for individuals with higher levels of alcohol identity and self-esteem. Direct persuasion is hypothesized to be less effective for individuals with higher levels of alcohol identity and self-esteem.

## Method

### Participants and design

Based on an a priori estimation of statistical power of (1-β) = .8 and an estimated effect size Cohen’s *f^2^ =* .15 (derived from Loman et al., 2018), a minimum of 150 participants was required for this experiment. Due to practical restraints one hundred and forty-nine participants were tested (97 women; 52 men) ranging in age from 18 to 34 years (*M* = 21.87, *SD* = 2.90). They participated in the experiment for course credit or a monetary reward of 5 euros. Participants were recruited at the University and were randomly assigned to one of five conditions in a 2 (persuasion-technique: self-persuasion vs. direct persuasion) x 2 (message exposure: long vs. short) between-subjects design with a control group (no persuasion). The dependent variable was grams of beer consumed during a fifteen-minute beer tasting task. The experiment was approved by the university ethics committee and written informed consent was obtained prior to the experiment.

### Procedure

The experiment consisted of two parts: (1) an online questionnaire and (2) a beer tasting session in a bar laboratory. Participants were recruited via the university’s research participation system and were eligible to participate if they were over 18 years old (the legal drinking age in the Netherlands) who indicated on the systems prescreen that they had consumed alcohol before (to exclude non-drinkers who would not be the target group of the intervention) and had a good understanding of the Dutch language. As a cover story they were told that the experiment was about taste perception. Participants were required to fill out an online questionnaire assessing self-esteem, alcohol identity and previous alcohol consumption frequency and intensity, a minimum of 24 hours before the beer taste test would take place.

The second part took place in an interaction room outfitted as a bar. Testing took place between 16:00 and 21:00 hours. Depending on the condition, either a self-persuasion or a direct persuasion anti-alcohol poster was displayed on one of the walls (posters adopted from [17]). In the long message exposure conditions, the posters were displayed on the wall behind the bar, directly in view of the participants during the beer taste task, which took approximately 15 minutes. In the short message exposure conditions, the poster was displayed on the wall opposite the bar (i.e., out of view, behind participants). In order to ensure successful exposure in the short message exposure conditions, all participants were asked to fill in demographic information (i.e., age, sex and education level) on a computer underneath the short message exposure poster location, which took approximately 60 seconds to complete.

Then, participants were asked to sit on a stool in front of the bar for the beer taste test. They were told that they would be tasting different brands of beer, and that they were required to fill out a (bogus) beer taste questionnaire. The real goal of the task was to measure how much beer they consumed during the test [50]. This taste-rating task was selected over a free-choice ad libitum drinking paradigm (as in [17]), because it does not influence normal consumption ([50]; also see [51]) but does ensure that all participants drink beer, thereby, increasing variance in the consumption data. Participants were asked to blindly taste three different popular brands of Dutch beer (i.e., Amstel, Grolsch, and Heineken, always in this order) served in 200 milliliter glasses (as in [52]). Participants were not told how many beers they would be tasting until they finished tasting the last one. After tasting each beer, participants were asked if they could guess the brand of the beer to further mask the real goal of the experiment. Finally, participants were thanked, rewarded, debriefed using a funnel procedure to check the poster exposure length manipulations, and dismissed.

### Materials and measures

#### Stimulus materials

**Fig 1:**
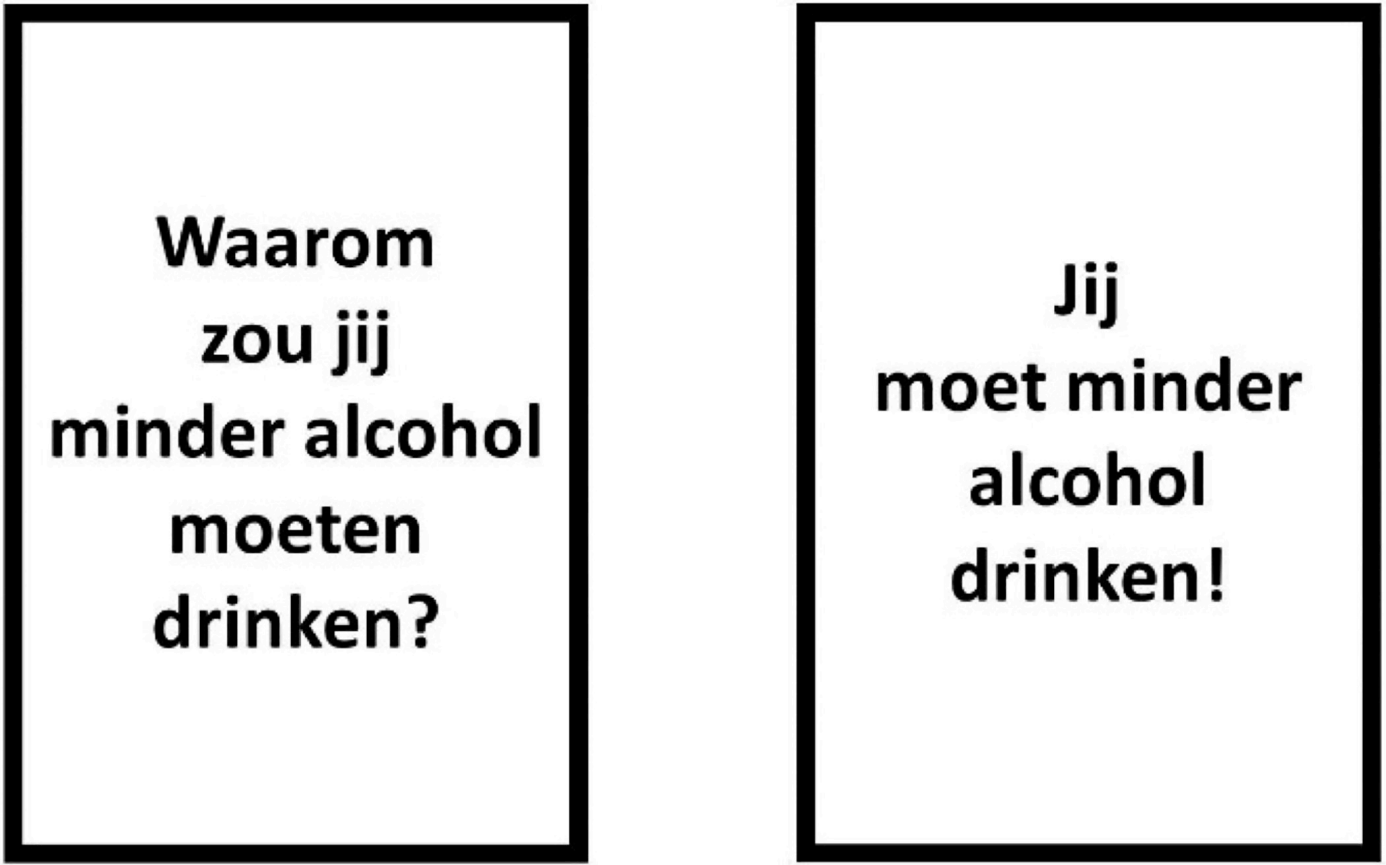
Stimulus materials in the experiment. Left is self-persuasion: “Why do you have to drink less alcohol?”; right is direct persuasion: “You have to drink less alcohol!”.

Participants were exposed to one of two anti-alcohol posters varying in persuasion-technique: (1) a self-persuasion version with the question “Why do you have to drink less alcohol?” or (2) a direct-persuasion version with the statement “You have to drink less alcohol!” (both translated from Dutch; see Fig 1; adopted from [17]). Note that the direct persuasion poster does not resemble a real-life anti-alcohol message, which typically states “drink responsibly” in the Netherlands. The choice for the direct persuasion message was made to compare the relative effectiveness of a clear statement to an open-ended question to establish a proof of principle. Both posters had an identical layout: A black frame against a white background with the message text centered both vertically and horizontally. The size of the posters was A2. It is noteworthy that translated to English the wording of the question might be interpreted as a promotion for drinking. However, this is very unlikely in the original Dutch wording, which is supported by the finding that nearly none of the participants generated counter-arguments to the question poster (see experiment 1 in [17]).

#### Beer consumption

The main outcome measure in this experiment was total beer consumption during the beer taste test in the bar lab. Participants tasted three 200 milliliter glasses of different beers with the same alcohol content (5%). The amount of beer consumed was calculated by subtracting the weight of the glass (in grams) after tasting from the weight before tasting. For two participants the amount of beer consumed was not registered and they were therefore not included in any of the analyses. A total score for each participant was calculated by adding up the three weight differences (*M* = 168.32, *SD* = 119.48).

#### Self-esteem

Self-esteem was measured with the Rosenberg Self-Esteem Scale [53]. The scale consists of ten statements (e.g., “On the whole, I am satisfied with myself”) to which participants indicated their agreement on a five-point scale ranging from 1 (*not at all agreed)* to 5 (*completely agreed*). For each participant, the mean over these ten items was calculated as an indication of general self-esteem, (Cronbach’s α = .89, *M* = 3.27, *SD* = .52). Appropriate items were reverse coded (see [53]) so that higher average scores reflect higher levels of self-esteem.

#### Alcohol identity

Alcohol identity was measured with five statements (e.g., “Drinking alcohol is an important part of who I am”) rated on a 7-point Likert scale ranging from 1 (*strongly disagree*) to 7 (*strongly agree*), adopted from [47]. For each participant, the mean over these five items was calculated as an indication of self-perceived alcohol identity (Cronbach’s α = .68, *M* = 3.61, *SD* = .99). Higher scores indicate a higher self-perceived alcohol identity.

#### Alcohol consumption frequency

In order to control for the effects of previous alcohol consumption behavior, frequency of alcohol consumption over the past four weeks was measured using four questions (one for each of the preceding four weeks; e.g., “On how many days did you drink alcohol in the past week?”; adopted from [54]). For each participant, the mean over these four items was calculated as an indication of the frequency of previous alcohol consumption (Cronbach’s α = .79, *M* = 2.35, *SD* = 1.09).

#### Alcohol consumption intensity

In order to control for the effects of intensity of previous alcohol consumption behavior, amount of alcohol consumed in the previous week was measured using four questions: during weekdays and in the weekend, inside and outside the home (e.g., “How many glasses of alcohol did you consume in the past week, during weekdays, at home?”; adopted from [55]). For each participant, the sum of these four items was calculated as an indication of intensity of previous alcohol consumption (*M* = 11.11, *SD* = 11.30).

#### Funnel debriefing

After the beer tasting task, participants were debriefed via a funnel procedure to check awareness of the goal of the experiment and the poster exposure length manipulation, expecting higher recall in the long exposure conditions as an indication of higher message elaboration. They were asked what they think the study was about, to which 12 participants (8%) referred in some way that volume of beer consumed might be of interest. Only 2 participants (1%) indicated that posters might be part of the experiment. Awareness of the study goals did not vary across conditions. From this we conclude that the cover story was largely succesful.

Participants were then asked whether they saw a poster in the room, whether they recalled the topic of the poster, and whether they could reproduce the exact wording of the poster. See Table 1 below for the responses per experimental condition. The number of people that had seen the poster differed significantly between the long- (*n* = 43 out of 59) and short-exposure (*n* = 22 out of 59) conditions (χ^2^ = 14.00, *p* < .001, *BF*_*10*_ = 533). The number of people that had seen the poster did not differ significantly between the self- (*n =* 30 out of 60) and direct persuasion (*n* = 35 out of 58) conditions (χ^2^ = .92, *p* = .34, *BF*_*01*_ = 2.31).

**Table 1:** Poster manipulation checks in the experimental conditions: Participants that indicated “no” or were incorrect.

Persuasion type:	Self-persuasion		Direct persuasion		Total
Exposure length:	short	long	short	long	
Did you see a poster in the bar?	15 of 28 (54%)	7 of 29 (24%)	21 of 30 (70%)	8 of 29 (28%)	51 of 116 (44%)
What was the topic of the poster?	17 of 28 (61%)	8 of 29 (28%)	23 of 30 (77%)	8 of 29 (28%)	56 of 116 (48%)
What was the exact wording of the poster?	20 of 28 (71%)	18 of 29 (62%)	25 of 30 (83%)	14 of 29 (48%)	77 of 116 (66%)

### Strategy of analysis

Before the main analyses, the randomization was checked and correlations between the control variables and beer consumption during the experiment were calculated. Then, to test if participants consumed more beer in the experimental conditions compared to the control condition an ANOVA was calculated with beer consumption as the dependent variable and the five condition as the between subject-factor. Next, to test the main effects of persuasion type and exposure length and their interaction, a linear regression was calculated with beer consumption as the dependent variable, persuasion type and exposure length as between-subject factors and sex, alcohol consumption frequency- and intensity as control variables. Factors were coded using sum-to-zero contrasts and covariates were mean-centered for interpretation purposes. The control condition was not included in this model, because it could not be scored on persuasion type or exposure length. Finally, to test for moderation of self-esteem and alcohol identity, the regression was repeated including both variables (also centered) as covariates and as interactions with persuasion type and message exposure. All frequentist analyses were conducted with R statistical software [56].

#### Bayes factors

In addition to p-values, Bayes Factors (BFs; [57,58]) are reported. BFs are the ratio between the likelihood of the data given one hypothesis (typically H1), and the likelihood of the data given another hypothesis (typically H0). For example, BF_10_ = 5 (or BF_01_ = 0.2) indicates that the data are five times as likely to occur under H1 than under H0. One of the advantages of using Bayes Factors is that they allow to distinguish between inconclusive data (e.g., BF_01_ = 1), and support for H0 (e.g., BF_01_ = 10). All Bayes Factors were calculated using JASP version 0.9.1 (2018), using default priors (i.e., a Cauchy distribution with width .707 for the Bayesian ANOVAs and the Bayesian t-test, and r scale covariates of .354 for the linear regression). The decision to report BFs was made post hoc.

## Results

### Randomization checks and descriptive statistics

Before conducting the main analysis, it was examined whether all non-experimental measures differed across conditions. Previous alcohol consumption frequency and intensity, sex, self-esteem, and alcohol identity did not significantly differ across conditions, indicating that the randomization procedure was successful (all *p* > .16). Table 2 depicts the means of these variables per condition.

**Table 2:** Sample means and standard deviations by condition.

Persuasion type	Self-persuasion		Direct persuasion		Control	Total
Exposure length	Short	Long	Short	Long		
	*n* = 29	*n* = 29	*n* = 30	*n* = 30	*n* = 31	*N* = 149
	*M* (*SD*)	*M* (*SD*)	*M* (*SD*)	*M* (*SD*)	*M* (*SD*)	*M* (*SD*)
Beer consumed (g)	158.79 (110.30)	200.41 (140.06)	170.13 (105.75)	162.48 (121.38)	149.41 (119.13)	168.32 (119.48)
Alcohol c. frequency	2.64 (1.21)	2.49 (1.27)	2.50 (1.09)	2.04 (.91)	2.13 (.93)	2.35 (1.09)
Alcohol c. intensity	12.22 (13.60)	11.00 (7.05)	12.43 (13.28)	9.86 (8.74)	10.13 (12.38)	11.11 (11.30)
Alcohol identity	3.43 (.81)	3.58 (1.00)	3.81 (1.07)	3.48 (1.10)	3.72 (.93)	3.61 (.99)
Self-esteem	3.20 (.48)	3.34 (.58)	3.39 (.55)	3.26 (.42)	3.15 (.55)	3.27 (.52)

There was a significant correlation between prior alcohol consumption frequency and beer consumption (*r* = .37, *p* < .001, *BF*_*10*_ = 1765), indicating that those who drank more frequently consumed more beer during the experiment (see Table 2 for corresponding statistics). Similarly, prior alcohol consumption intensity was positively correlated with beer consumption (*r* = .30, *p* < .001, *BF*_*10*_ = 48). Sex also had an effect on drinking behavior, Welch’s *t*(90.20) = -5.50, *p* < .001, *BF*_*10*_ > 10,000, indicating that men (*M* = 238.41 *SD =* 118.70) consumed more beer than women (*M* = 130.30, *SD* = 101.86). Because the three control variables were significantly related to the main outcome variable beer consumption, they are included in the regression analysis to reduce unexplained error variance, allowing to more accurately assess the effects of the independent variables [59]. A significant effect of experiment leader on beer consumption was found, *F*(2, 142) = 3.61, *p* = .03, however *BF*_*01*_ = 1.757, suggesting that beer consumption might differ depending on who led the experiment (*M1* = 134.41, *SD* = 100.48; *M2* = 189.67, *SD* = 130.76; *M3* = 183.84, *SD* = 107.27). Adding experiment leader in the analyses does not change the results and was therefore omitted.

### Beer consumption

First, a one-way ANOVA with beer consumption as the dependent variable and condition (control, self-persuasion short, self-persuasion long, direct persuasion short, and direct persuasion long) as the independent variable was conducted using sum-to-zero contrast coding. There was no significant effect of condition on beer consumption *F*(4, 140) = .76, *p* = .55, *BF*_*01*_ = 14.31, indicating that none of the experimental conditions differed significantly from each other in terms of beer consumption.

Next, the linear regression with beer consumption as the dependent variable, persuasion type, exposure length and their interactions, as well as sex and previous alcohol consumption frequency and intensity as independent variables yielded no significant effects for the experimental manipulations nor their interaction (see Table 3). This indicates that participants’ beer consumption was unaffected by the posters, regardless of exposure length. Significant effects for sex and previous alcohol consumption frequency were found, indicating that (1) men consumed more beer than women, and (2) participants who consumed alcohol more often, consumed more beer during the experiment.

**Table 3:** Linear regression analysis: Standardized regression coefficients predicting grams of beer consumed.

Variable	*b*	*SE*	*R*^*2*^	*p*	*BF*_01_*
Step 1			.31		
Persuasion type	2.53	8.35		.76	4.16
Exposure length	3.94	8.42		.64	4.11
Persuasion type x exposure length	-10.80	8.26		.19	15.12
Sex	**107.27**	**18.71**		< .001*	
Alcohol c. frequency	**20.07**	**9.27**		.03*	
Alcohol c. intensity	-.26	.95		.78	
Step 2			.29		
Persuasion type	-1.17	10.29		.91	4.16
Exposure length	9.37	10.40		.37	4.11
Persuasion type x exposure length	-7.78	9.06		.39	15.12
Sex	**100.44**	**20.51**		< .001*	
Alcohol c. frequency	13.22	10.30		.20	
Alcohol c. intensity	-.81	1.13		.48	
Self-esteem	14.81	19.22		.44	4.16
Alcohol identity	20.68	12.01		.09	1.03
Persuasion type x self-esteem	12.36	21.25		.56	15.39
Persuasion type x alcohol identity	-.11	10.68		.99	4.09
Exposure length x self-esteem	-8.97	21.18		.67	15.09
Exposure length x alcohol identity	6.90	10.66		.52	3.86
Self-esteem x alcohol identity	.81	22.11		.97	2.81
Persuasion type x exposure length x self-esteem	-5.12	19.99		.80	50.63
Persuasion type x exposure length x alcohol identity	-3.82	10.68		.72	13.89
Persuasion type x self-esteem x alcohol identity	7.68	22.89		.74	10.28
Exposure length x self-esteem x alcohol identity	31.08	21.87		.16	9.48

Significant results in bold.

* *p* < .05

** *p* < .001.

The BFs represent the ratio between the explanatory value of a null-model including only alcohol consumption frequency, alcohol consumption intensity, and sex, calculated against a model including the effect for which the BF is given.

Finally, repeating the regression including the moderators self-esteem and alcohol identity yielded a significant effect for sex only, and no main or interaction effects for self-esteem and alcohol identity. The previously significant effect for alcohol consumption frequency was no longer significant in this model.

### Explorative analyses

A large number of participants (44%) in the experimental conditions indicated in the exit interview that they did not recall seeing a poster. Therefore, it was decided to exploratively repeat all main analyses for the subsample of only participants who recalled seeing the experimental poster. To increase the power of these analyses, the short and long exposure length groups were collapsed.

#### Randomization checks and descriptive statistics for the subsample

Before conducting the explorative analysis, it was examined whether all non-experimental measures differed across conditions. For previous alcohol consumption frequency a significant difference was found between conditions, *F*(2, 90) = 3.24, *p* = .04. However, Tukey post hoc comparisons indicated no significant differences between any of the conditions (all *p* > .06). Previous alcohol consumption intensity, sex, self-esteem, and alcohol identity did not significantly differ across conditions, indicating that the randomization procedure was successful for these variables (all *p* > .14). Table 4 depicts the means of these variables for subsample of participants who recalled seeing the experimental poster by persuasion type.

**Table 4:** Sample means and standard deviations by persuasion type for the subsample of participants who recalled seeing the experimental poster.

Persuasion Type	Self-persuasion	Direct persuasion	Control	Total
	*n =* 35	*n =* 30	*n =* 31	*n* = 96
	*M* (*SD*)	*M* (*SD*)	*M* (*SD*)	*M* (*SD*)
Beer consumed (g)	184.06 (140.33)	146.03 (91.02)	149.41 (119.13)	161.23 (119.87)
Alcohol c. frequency	2.69 (1.25)	2.08 (.94)	2.13 (.93)	2.30 (1.08)
Alcohol c. intensity	12.63 (11.87)	12.07 (13.37)	10.13 (12.37)	11.62 (12.45)
Alcohol identity	3.79 (.85)	3.56 (1.14)	3.72 (.93)	3.69 (.97)
Self-esteem	3.35 (.49)	3.25 (.56)	3.15 (.55)	3.25 (.53)

As in our full sample, there was a significant correlation between both prior alcohol consumption frequency (*r* = .47, *p* < .001, *BF*_*10*_ = 5503) and intensity (*r* = .32, *p* < .001, *BF*_*10*_ = 13), and beer consumption. Here, too, there was a sex difference in beer consumption, Welch’s *t*(51.51) = -4.28, *p* < .001, *BF*_*10*_ = 783, indicating that men (*M* = 234.57 *SD =* 117.64) consumed more beer than women (*M* = 126.86, *SD* = 105.26). Therefore, these three control variables were all included in the regression analyses for this subsample. The effect of experiment leader on beer consumption was nonsignificant in the subsample, *F*(2, 91) = 1.60, *p* = .21, *BF*_*01*_ = 2.55.

#### Beer consumption for the subsample

First, a one-way ANOVA with beer consumption as the dependent variable and condition (control, self-persuasion and direct persuasion) as the independent variable was conducted using sum-to-zero contrast coding. There was no significant effect of condition on drinking behavior *F*(2, 91) = 1.02, *p* = .37, *BF*_*01*_ = 5.54, indicating that none of the experimental conditions differed significantly from each other in terms of beer consumption.

Next, the linear regression with beer consumption as the dependent variable, persuasion type, sex and previous alcohol consumption frequency and intensity as independent variables yielded no significant effects for the experimental manipulations nor their interaction (see Table 5). This indicates that participants’ beer consumption was unaffected by the posters. Significant effects for sex and previous alcohol consumption frequency were found, indicating that (1) men consumed more beer than women, and (2) participants who consumed alcohol more often, consumed more beer during the experiment.

**Table 5:** Linear regression analysis: Standardized regression coefficients predicting grams of beer consumed for the subsample of participants who recalled seeing the experimental poster.

Variable	*b*	*SE*	*R*^*2*^	*p*	*BF*_01_*
Step 1			.38		
Persuasion type	9.11	11.70		.44	2.78
Sex	**120.58**	**25.66**		< .001**	
Alcohol c. frequency	**37.53**	**12.98**		< .01*	
Alcohol c. intensity	-1.21	1.18		.31	
Step 2			.41		
Persuasion type	10.67	12.31		.39	10.23
Sex	**105.80**	**26.73**		< .001**	
Alcohol c. frequency	25.71	13.33		.06	
Alcohol c. intensity	-2.03	1.33		.13	
Self-esteem	26.48	24.14		.28	11.77
Alcohol identity	**43.56**	**15.34**		< .001**	1.00
Persuasion type x self-esteem	-8.27	24.20		.73	29.96
Persuasion type x alcohol identity	-15.78	12.77		.22	2.49
Self-esteem x alcohol identity	-25.29	22.28		.26	3.11
Persuasion type x self-esteem x alcohol identity	6.05	23.25		.80	6.80

Significant results in bold.

* *p* < .01

** *p* < .001.

The BFs represent the ratio between the explanatory value of a null-model including only alcohol consumption frequency, alcohol consumption intensity, and sex, calculated against a model including the effect for which the BF is given.

Finally, repeating the regression including the moderators self-esteem and alcohol identity yielded a significant effect for sex and alcohol identity, and no main or interaction effects for alcohol consumption intensity and self-esteem. This indicates that men consumed more beer than women and that participants for whom alcohol is more important for their identity, also consumed more beer in the experiment. The previously significant effect for alcohol consumption frequency was no longer significant in this model.

## Discussion

The primary aim of the current experiment was to test the effectiveness of anti-alcohol posters framed as statements (direct persuasion) or open-ended questions (self-persuasion) to reduce alcohol consumption in a beer taste test, under conditions of short- (low message elaboration) or long (high message elaboration) message exposure, compared to a control condition without a poster. Results indicated that both posters failed to affect alcohol consumption in a beer taste test, regardless of exposure length, and that this was independent of participant’s self-perceived alcohol identity and self-esteem.

In line with previous work, direct persuasive anti-alcohol posters did not affect alcohol consumption. Similarly, experiments on self-persuasion in media messages specifically [17] and media effects research for alcohol in general [15] yielded little or no support for effectiveness of direct persuasion. However, the finding that the self-persuasive anti-alcohol posters failed to change consumption behavior is not in line with previous self-persuasion research. That is, recent experiments on self-persuasion in media messages showed positive (albeit small) behavioral change effects [17,20]. In the current experiment neither poster had a significant effect on the drinking behavior of the participants.

A possible explanation for the unexpected null-findings in this research could be that only 56% of the participants in the experimental conditions recalled seeing the posters, with even smaller percentages being able to correctly recall what the poster was about and what the exact wording of the posters was. Research has shown that responsible drinking messages are poorly attended in an environment rich in drinking cues, such as a bar [9]. It is possible therefore, that no persuasion effects were found because a large number of participants did not see the poster. Future research could address this issue with manipulation checks that objectively measure exposure, for example by registering whether and how long participants actually look at the posters using hidden cameras to clarify whether and how attention time influences drinking behavior.

We argue, however, that not having seen the posters is likely not the main reason for the null-effects in the current experiment for two main reasons. First, low recall of the posters in the exit interview does not mean the posters had no effect on behavior as messages can have effects without explicit attention to or awareness of the persuasive communication (e.g., [60–63]; also see [41]). Second, the explorative analyses among only the participants who did explicitly recall seeing the posters did not yield significant effects of the manipulations. We therefore deem it more likely the posters were simply ineffective to change alcohol consumption behavior in the experimental setting we created.

Two other, related yet distinct explanations seem appropriate for the null findings in the experiment. Both spring from methodological choice for a beer-taste test paradigm in the current experiment, which *requires* participants to drink, versus a free choice paradigm used in other studies. Specifically, when individuals could choose to drink [17] or smoke [20] consumption was reduced after being exposed to a self-persuasive media message. In the current experiment this freedom to choose was not possible: all participants committed to consume alcohol by enrolling in the study. As a direct consequence, participants might (1) have considered the anti-alcohol posters to not be applicable to them in this particular setting, because after all they were required to drink alcohol for the experiment. The individuals could therefore have thought that the poster was irrelevant for them instead of thinking of reasons why they should drink less alcohol, rendering it ineffective regardless of exposure length.

Alternatively (2), the participants’ sense of agency over their alcohol consumption might have been reduced, which has been shown to decrease the effectiveness of self-persuasion techniques [32]. In other words, because participants did not feel they could control their alcohol consumption behavior (they already agreed to drinking), self-persuasion did not occur. Notably, the study by Damen and colleagues also showed increased effectiveness of direct persuasion techniques under conditions of a reduced sense of agency. This effect was not found in the current experiment. The role of perceived agency over the target behavior in self-persuasion media interventions therefore, is unclear at this point and should be taken into account in future self-persuasion experimentations.

The finding that exposure length did not influence the effectiveness of either persuasion type to change alcohol consumption was unexpected in light of elaboration research [34,64]. It does make sense, however, if both posters are unable to affect alcohol consumption because they were perceived as irrelevant or due to reduced experienced agency of the participants over their alcohol consumption as described above.

Two limitations pertaining to the poster exposure manipulation should be noted. First, it was assumed that longer message exposure would increase message elaboration and, thus, would result in more generated arguments ‘why to drink less alcohol’ in the self-persuasion conditions. However, this assumption was not explicitly measured. Second, it would be possible that participants in the short exposure conditions elaborate about the messages even after exposure. Future research would do well to address these possibilities for example with a think-aloud or thought-listing task [65] respectively during or following a short versus long self-persuasion poster exposure. Alternatively, instead of manipulating the length of exposure to the posters, a cognitive load manipulation could be selected. The advantage of this would be that elaboration about the message should not be possible under high cognitive load. Both suggestions would increase internal validity of the results at the cost of ecological validity.

A noteworthy difference of the current experiment compared to previous studies is that during recruitment participants were told that they would be drinking beer for the experiment. This could have resulted in a selection bias, attracting individuals that were interested in alcohol. Even though this concern is not really evident in the alcohol identity measures in the experiment, which reflect ‘average’ importance of alcohol for the participants (see Table 1), future research could address this by briefing participants about the contents of the experiment after recruiting (but before the experiment starts to allow participants to withdraw from participating). This way possible selection bias could be diminished or at the very least percentages of withdrawal could be given as an indication of the size of the bias.

Two final limitations of the current experimental setup should be noted. First, it was chosen to have participants only consume alcohol in the beer taste test. For future research, however, it would likely be better to offer both alcoholic and non-alcohol beverages to the participants as this will reduce thirst as a confound [66]. Second, participants Breath Alcohol Concentrate was not measured upon entering the bar laboratory. It would be advisable for future research to include this measurement as an exclusion criterion for participation, as alcohol consumption prior to the beer taste test might affect consumption in the test.

### Implications

The current findings might have important implications for self-persuasion research. Specifically, it is possible that ‘no-choice’ paradigms lead to systematic underestimation of the effectiveness of self-persuasion in media messages to change behavior. The limited number of studies to date indeed have shown significant differences in free-choice paradigms for alcohol consumption [17] and smoking [20], but are not found when the freedom to choose is diminished in the measurement task, as happened in the current experiment. Future research should prioritize testing the impact of (reduced) freedom to choose on the effectiveness of self-persuasive media messages, for example by directly comparing the effects of direct- and self-persuasion posters in an ad libitum drinking task with the effects in a forced drinking task such as the beer taste test used in the current experiment.

If indeed self-persuasion is only effective when individuals experience full freedom to choose their behavior, this should not be a problem for real life interventions because choice freedom is generally untampered with there (as opposed to laboratory tasks). However, the finding might have important consequences for how self-persuasion effects are researched. Specifically, forced-choice measurement tasks should be avoided. Concerning self-persuasion techniques to reduce alcohol consumption on a global scale, the current findings add that behavioral self-persuasion effects triggered by media messages are likely very small. This raises the question if it is really helpful to use self-persuasion strategies in mass media. Realistically, it will not reduce consumption even close to what is needed to put a dent in alcohol’s contribution to global disease and mortality. Still it should be noted that positive effects in certain situations are found in a relatively new and growing line of experimentation [17–20,26,67]. In these studies self-persuasion has consistently outperformed direct persuasion counterparts and no persuasion controls. Large-scale application, therefore, might still yield tangible benefits, and at the very least self-persuasion seems more effective than direct persuasion. Further testing seems appropriate.

### Conclusion

In sum, the current experiment shows that alcohol consumption in a beer taste test is not affected by anti-alcohol posters using self-persuasion- or direct persuasion techniques under conditions of both high- and low message elaboration. Although these findings are surprising in the light of previous self-persuasion research, they point towards an important possible mediator for self-persuasive media messages to be effective: the role of freedom to choose or perceived agency over the target behavior. Specifically, it is possible that self-persuasion only occurs when individuals can freely choose to engage in the target behavior. Future research should prioritize examining this idea, as it could have important consequences for the way self-persuasion is researched.
